# Lived Experiences of Women with Arteriovenous Fistula Undergoing Hemodialysis: A Phenomenological Study

**DOI:** 10.3390/healthcare14101296

**Published:** 2026-05-11

**Authors:** Bo Min Kim, Jin Ah Kim

**Affiliations:** College of Nursing, Dongguk University WISE Campus, Dongdaero 123, Gyeongju-si 38066, Republic of Korea

**Keywords:** arteriovenous fistula, body image, hemodialysis, psychosocial adaptation, qualitative research, women’s health

## Abstract

**Background/Objectives:** An arteriovenous fistula (AVF) is the preferred vascular access for hemodialysis (HD). Beyond its clinical function, an AVF creates visible bodily changes that may affect identity, social positioning, and psychological adaptation. Women undergoing HD via an AVF may perceive these changes in relation to sociocultural expectations surrounding body image and gender roles. This study explored the lived experiences of women undergoing HD via an AVF and considered the implications for AVF management. **Methods:** Using Colaizzi’s descriptive phenomenological method, in-depth interviews were conducted with nine women undergoing regular HD via an AVF in South Korea. The data were analyzed to identify essential themes and the fundamental structure of their experiences. **Results:** The essential structure was described as “women’s lives shaken and repositioned amid visible, life-sustaining bodily changes.” Three interrelated theme clusters emerged: shaken self-confrontation of the visible body; being repositioned within the relational world; and acceptance formed within the polarity of life and threat. The visibility of the AVF influences identity, autonomy, and social interactions. **Conclusions:** AVF management should extend beyond technical maintenance and include psychosocial assessments and sex-sensitive supportive strategies. Addressing the visible and relational dimensions of the AVF may enhance the quality and safety of HD care.

## 1. Introduction

Chronic kidney disease (CKD) is defined as an abnormality in kidney structure or function that lasts for at least three months [1]. The Global Burden of Disease Study 2023 reported an age-standardized global prevalence of CKD among adults of 14.2% [2], whereas 6.3% of adults in Korea were diagnosed with CKD in 2021 [3]. These data indicate that CKD constitutes a major public health concern, calling for coordinated management at both the national and global levels.

When CKD progresses to its end stage, kidney replacement therapy (KRT) is necessary for survival. KRT is categorized into hemodialysis (HD), peritoneal dialysis, and kidney transplantation [4]. Globally, approximately 69% of patients undergoing KRT undergo HD [5]. In Korea, approximately 78% of patients undergo HD [6], establishing it as the most common and central treatment method for end-stage renal failure.

A vascular access site that provides continuous and sufficient blood flow is essential for the stable implementation of HD. According to domestic statistics, approximately 78% of patients undergoing HD use autologous arteriovenous fistulas (AVFs), whereas arteriovenous grafts and tunneled catheters account for 15% and 5%, respectively [7]. AVFs display superior long-term patency rates and lower infection risks, making them a preferred clinical vascular access method [8]. However, AVFs can cause structural changes such as intimal hyperplasia and vascular dilatation during repeated punctures and long-term use [9]. While these changes result from physiological and structural adaptations to repeated punctures and increased blood flow, vessel dilation and protrusion can lead to visible bodily changes. These changes can affect how patients recognize and understand their bodies, leading to body image distortion or dissatisfaction. Such changes can result in psychological and social difficulties, including feelings of depression, reduced self-efficacy, and social withdrawal [10].

It has been suggested that the psychological burden may be relatively more pronounced in women undergoing HD. A study conducted in Iran found that female patients exhibited higher levels of concern and anxiety regarding physical changes than male patients, and significant associations were observed between body image perception and self-management behaviors [11]. This evidence indicates that the physical changes that occur during treatment may have stronger consequences for women.

These phenomena require insight beyond the individual level and within a sociocultural context. A survey of American adults found that “physical attractiveness” was a key trait for women [12]. Similarly, in Korean society, idealized female images are repeatedly reproduced through the media, shaping societal expectations regarding women’s appearances and bodies [13]. Within this cultural context, treatment-induced physical changes may affect an individual’s identity and social perceptions beyond functional impairment alone. Moreover, the relatively high prevalence of CKD in women underscores the need for a more in-depth review of these issues. According to Global Burden of Disease 2023 data, CKD prevalence is higher in women than in men [2], and United States data for 2017–2020 similarly reported a higher CKD prevalence among women than men [14]. These results emphasize the need for a careful exploration of the psychological and social significance of bodily changes experienced by women with CKD during treatment. However, current HD patient management primarily focuses on physiological and technical aspects, such as maintaining AVF function and preventing complications. Body image changes resulting from the AVF and associated psychological and social adaptation issues have received insufficient attention, particularly for women. Despite the possibility that these physical changes affect their self-identity and relational positioning, sex-sensitive management strategies that respond to these concerns remain limited. Given that negative perceptions of body image can affect self-management behaviors, therapy compliance, and social support engagement, body image is an essential domain that is both directly and indirectly tied to the quality and safety of patient care, extending beyond individual experiences.

This gap in management aligns with prior research. Previous studies primarily focused on clinical indicators such as vascular access maintenance [15], complication management [16], and dialysis efficiency [17]. In-depth examinations of how women with an AVF perceive their altered bodies and how they experience themselves, others, and social relationships through their bodies are relatively scarce. Understanding the subjective meanings of these experiences and how they exist within the lifeworld requires a phenomenological approach. Thus understanding the AVF experience of women undergoing HD can provide an important academic and clinical foundation for developing more holistic and integrated patient management strategies and guiding interventions.

Therefore, this study applied Colaizzi’s phenomenological research approach [18] to explore the lived experiences of women who underwent AVF formation for HD. It aimed to clarify how changes in body image shape individual psychological adaptation and social relationships and to offer foundational data for developing nursing interventions and social support strategies intended to enhance the quality of life of women undergoing HD.

## 2. Methods

### 2.1. Study Design

This study applied Colaizzi’s descriptive phenomenological approach to explore the lived experiences of women who underwent AVF formation for HD [18]. Descriptive phenomenology aims to disclose the essential structure of phenomena experienced by research participants. The researcher sought to derive meaning based on the participants’ statements while bracketing preconceptions as much as possible. This study explored how physical experiences related to AVF shape meaning within the context of women’s psychological adaptations and social relationships.

### 2.2. Study Population

The research participants were women undergoing regular HD at a general hospital’s artificial kidney center and a specialized kidney dialysis hospital located in Gyeongju, Gyeongbuk Province, South Korea. Participant selection criteria were as follows:

(1) Women with an autologous AVF created for HD following CKD diagnosis.
(2) Women who had undergone regular HD at least twice a week for over six months.
(3) Women who were able to verbally express their experiences and provided informed consent to participate in interviews.

Ten participants were recruited using purposive sampling. One participant was unable to complete the interview for health reasons; therefore, the analysis included data from nine participants. Data collection was terminated when saturation was deemed to have been reached.

### 2.3. Data Collection

Data collection took place over approximately three months, from April to June 2025. Data were collected through semi-structured, in-depth interviews. The interview questions were developed based on a review of the relevant literature and the study objectives. The core research question was: “After forming an AVF, how do women undergoing HD experience their bodily changes, and how is this experience constructed in terms of meaning within their daily lives and social relationships?” Open-ended and exploratory follow-up questions were used concurrently to explore the participants’ experiences.

One to three interviews were conducted with each participant, lasting 60–120 min per session. Interviews were conducted in a hospital counseling room or a quiet space where the participants felt comfortable. All interviews were recorded with the participants’ consent and transcribed. If new meanings or further explanations were required during the interview process, follow-up interviews were conducted to supplement the content. Data collection continued until data saturation was achieved. Data saturation was determined when no new significant statements, codes, or themes emerged and the existing thematic structure was sufficiently developed. More specifically, saturation was observed after the eighth interview, as no new conceptual categories were identified: the ninth interview confirmed the stability and completeness of the existing themes.

### 2.4. Data Analysis

**Figure 1:**
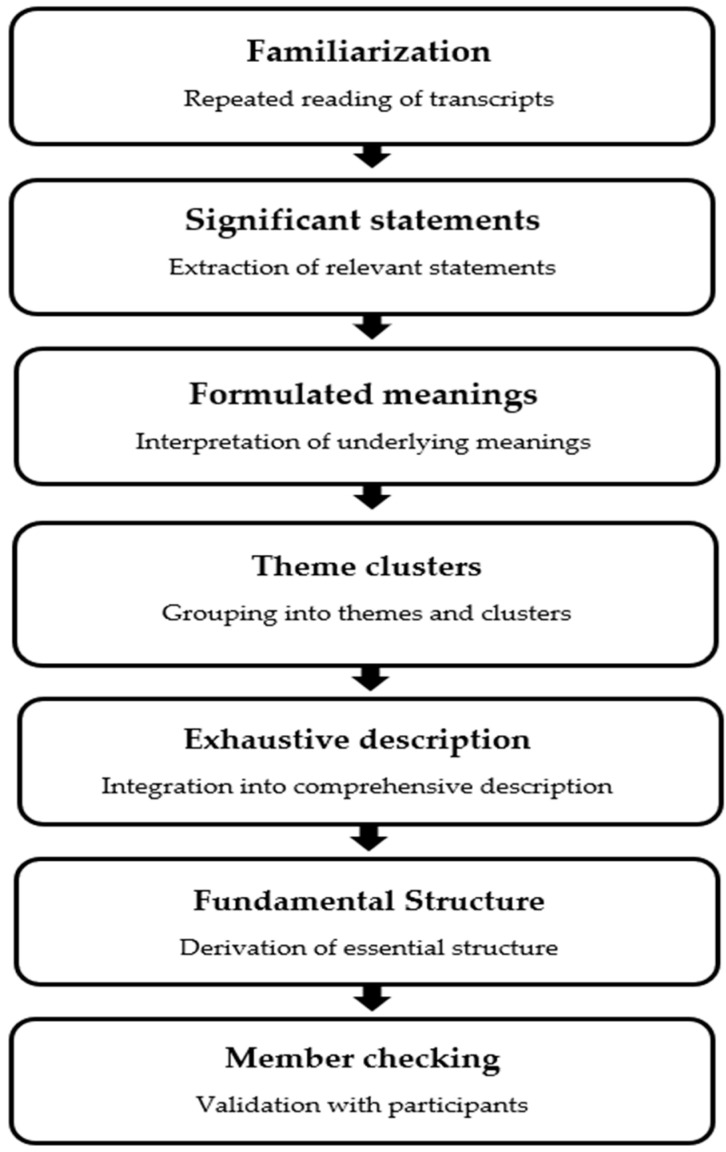
Data analysis process following Colaizzi’s descriptive phenomenological method.

Data were analyzed following the seven-step descriptive phenomenological procedure proposed by Colaizzi (1978) [18]. First, the transcribed interview data were read repeatedly to gain an overall understanding of the general context and atmosphere of the participants’ experiences. Subsequently, meaningful statements directly related to the research phenomenon were selected, and the meaning inherent in each statement was derived as a formulated meaning. The meanings obtained were compared and reviewed, grouped by similarity, and categorized into themes and theme clusters. These themes were further integrated to describe the structural meaning of the phenomenon experienced by the participants, eventually yielding a fundamental structure that explained the experiences of the women who underwent AVF formation. To ensure the validity of the analysis, the results obtained were presented to some participants to verify their correspondence with their experiences (member checking) (Figure 1).

All data were analyzed manually without the use of qualitative data analysis software. This approach is consistent with phenomenological research, which emphasizes close and iterative engagement with the data to capture the depth and meaning of participants’ lived experiences.

The primary researcher of this study was a nurse working in nephrology with experience in caring for HD patients; this clinical background served as a foundation for deepening the understanding of the research phenomenon. Simultaneously, to identify potential biases arising from prior perceptions during data interpretation, the researcher engaged in reflexive reflection through a research journal throughout data collection and analysis. Furthermore, the validity of the theme derivation and interpretation was verified through a review and iterative discussion with a researcher experienced in qualitative research.

The initial coding and analysis were conducted by the primary researcher, and the results were continuously reviewed and refined through iterative discussions with a co-researcher experienced in qualitative research. This collaborative process enhanced the credibility and rigor of the analysis by enabling critical reflection, comparison, and consensus-building throughout the analytical process.

A schematic representation of the data analysis process is presented in Figure 1, and an example of the analytical process from significant statements to themes is provided in Supplementary Table S1.

In this study, data saturation was confirmed through iterative comparison of the data, continuous reflection, and verification of thematic completeness. Given the nature of phenomenological analysis, frequency-based quantification of code repetition was not applied; instead, saturation was determined based on the absence of new meanings and the consistency of the thematic structure.

### 2.5. Research Rigor

This study aimed to ensure credibility, transferability, dependability, and confirmability in accordance with the evaluation criteria for qualitative research proposed by Lincoln and Guba [19].

First, member checks were conducted to improve credibility. This involved presenting themes and interpretations derived from the analysis to participants to confirm whether the content faithfully reflected their experiences. Peer debriefing was conducted continuously with peer researchers experienced in qualitative research throughout the research process. This study aimed to identify potential interpretive biases and maintain consistency in the analysis.

To ensure transferability, the research context, participants’ general characteristics, and data collection and analysis procedures are described in detail. This will enable readers to assess whether the research findings could be applied to similar situations.

Dependability was ensured via systematic recording and documentation of the entire process, from the research design through data collection, analysis, and theme extraction. These records were maintained as audit trails, enabling the verification of the rational coherence of the research process.

Finally, to ensure confirmability, the researcher maintained a research journal throughout the analysis and continuously reflected on preconceptions and interpretations. This ensured that the research findings were derived from participant statements and data rather than from the researcher’s personal perspective.

### 2.6. Ethical Considerations

This study was approved by the Institutional Review Board of Dongguk University WISE Campus (IRB No. 20250007). Before the commencement of the study, participants received a thorough explanation of the research purpose, procedures, interview content, and methods of data use and storage. Written informed consent was obtained from all participants. It was clearly communicated that participation was entirely voluntary and that participants could withdraw at any time during the research process without facing any disadvantages. The interviews were conducted anonymously, and the collected data were coded to prevent participant identification.

All recordings and transcripts were used solely for research purposes. Participants were informed that the materials would be kept safely and disposed of in accordance with the relevant regulations upon completion of the study. Considering the potential for psychological discomfort or emotional difficulty during the interview process, procedures for accessing counseling support were also explained.

## 3. Results

### 3.1. Participant Characteristics

The study included nine women undergoing HD via AVF. Participants ranged in age from 46 to 79 years. Most participants were widowed, divorced, or married, and the majority had completed middle or high school education. Most were unemployed, and the year of HD initiation varied widely, from 2000 to 2023 (Table 1).

### 3.2. Overview of the Essential Structure

**Figure 2:**
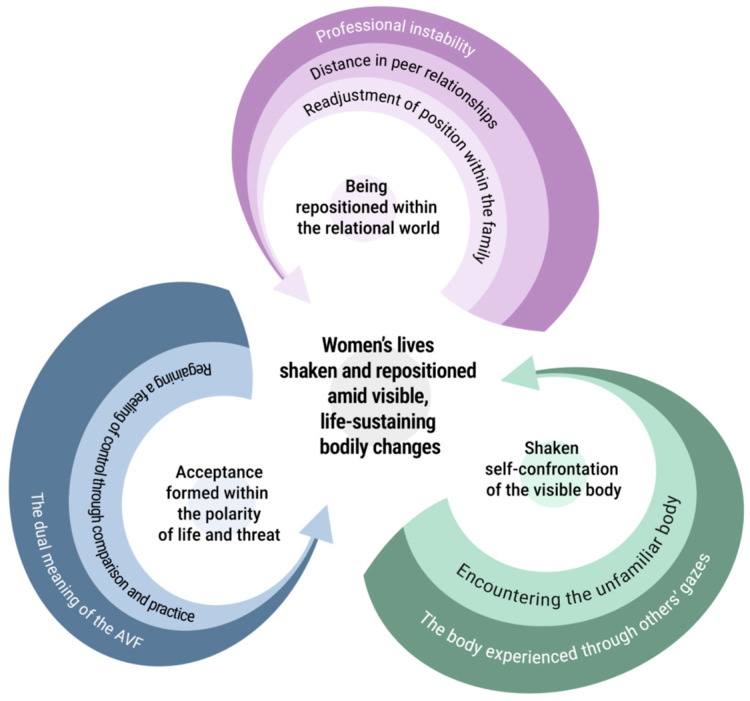
Conceptual Model of the Essential Structure of Women’s Lived Experiences Following AVF Formation for Hemodialysis.

This study explored the lived experiences of women who underwent AVF formation for HD. Data analysis showed that participants’ experiences converged into the core theme of “women’s lives shaken and repositioned amid visible, life-sustaining bodily changes.” This signifies that the visible bodily changes resulting from the AVF exceeded mere clinical outcomes, affecting self-perception and position within relational worlds, and were ultimately experienced as a process of reinterpreting and readjusting one’s existence. This core theme comprised three thematic clusters: (1) shaken self-confrontation of the visible body, (2) being repositioned within the relational world, and (3) acceptance formed within the polarity of life and threat (Table 2). To further illustrate the structural relationships among the core theme and its thematic clusters, a conceptual model was developed (Figure 2). The model visually represents the dynamic and cyclical process through which women’s experiences evolve, showing how visible bodily changes initiate self-confrontation, lead to relational repositioning, and ultimately contribute to a process of acceptance within the dual meaning of life and threat.

### 3.3. Shaken Self-Confrontation of the Visible Body

The bodily changes caused by an AVF are not exclusively a result of treatment; they represent an ongoing experience of confronting a visibly transformed body. Repeatedly observed bodily changes disrupted the familiar self-image, leading participants to recognize themselves as different from their “previous selves.” These visible changes triggered internal emotional turmoil while simultaneously making them conscious of others’ gazes, thereby forcing a reinterpretation of the body within a social context.

#### 3.3.1. Encountering the Unfamiliar Body

After AVF formation, participants no longer perceived their bodies as continuous with their former selves. The suddenly swollen arm and protruding blood vessels were perceived not only as a result of treatment but also as a visible marker of the disease taking a tangible form in the body. This marker was repeatedly recognized during daily activities, such as looking in the mirror or showering, leading to a shift in the familiar self-image. Through their altered bodies, participants became aware of a present self distinct from their “former self,” and this strangeness led to feelings of anxiety and loss. In other words, the AVF emerged as an experience that changed their perception of their bodies and their understanding of themselves.

“When I first saw it, it felt so incredibly unfamiliar because it wasn’t my own body. Having just one limb dangling as that made it even stranger… I was suddenly overcome with anxiety, wondering if this were something I’d have to live with forever”.

(P3, 51 years)

“Every morning and evening when I shower, I can’t help but see it, even if I try not to… ‘Do I have to live like this?’ I think. You can’t help feeling that sense of loss”.

(P1, 57 years)

#### 3.3.2. The Body Experienced Through Others’ Gazes

Participants did not perceive the AVF as a purely personal bodily change but rather as a part of a “social body” that is constantly interpreted through others’ gazes. The AVF formed on the exposed arm became a visible marker that was easily seen in public spaces. This compelled the participants to view their own bodies from others’ perspectives. They adjusted their daily behaviors by wearing long-sleeved garments or arm sleeves year-round and avoiding settings where bodily exposure was anticipated. Concealment and avoidance were social coping strategies to preemptively block the possibility of being defined or misunderstood as a diseased individual. In other words, the AVF functioned as a social marker that required management beyond the “visible body,” anticipating others’ judgments and stigma.

“I want to hide it… I never show this anywhere. Even if I want to wear short sleeves in summer, I can’t. I always wear long sleeves”.

(P1, 57 years)

“I don’t use public bathhouses. I’m embarrassed… People stare like I’m some kind of female gangster… I pretend not to care, wash quickly, and rush into the changing area”.

(P5, 56 years)

### 3.4. Being Repositioned Within the Relational World

The physical changes caused by the AVF compelled participants to reposition themselves within their families, peer groups, and occupational spheres, extending beyond the personal domain. Participants found it difficult to perform their roles, leading them to feel that they were repositioning their place in the relational world.

#### 3.4.1. Readjustment of Position Within the Family

After AVF formation, participants noticed changes in their roles at home. Their children and spouses perceived the AVF as something that required protection, limiting the participants’ actions or taking over roles on their behalf. This extended beyond simple physical care, becoming an experience that altered how they performed their roles as mothers and wives. The visible AVF marker led family members to perceive the participant as someone who required caution, resulting in a different approach to fulfilling family roles.

“My children say I shouldn’t do things. They say I shouldn’t lift heavy things… When I tried to shovel, they made a fuss. They said I shouldn’t do it”.

(P7, 46 years)

“After getting the AVF, my husband won’t allow me to do anything. He does all the laundry and cooking… He says I need to take care of my arm”.

(P4, 70 years)

#### 3.4.2. Distance in Peer Relationships

Within women’s peer groups, the AVF served as a visible marker of difference, creating subtle tension and distance. Repeated questions, stares, and awareness of gossip led participants to reexamine their bodies through others’ evaluations. This sometimes led them to reduce their participation in group activities and avoid relationships. It manifested as an experience of adjusting relationship dynamics while remaining conscious of others’ gazes.

“My friends stare at me for quite a long time… They ask, ‘Why is yours like that?’ Later, I saw them going off to talk quietly among themselves”.

(P1, 57 years)

“Honestly, even I think my arm looks a bit ugly… That’s why I don’t meet up. I just naturally drifted away”.

(P1, 57 years)

#### 3.4.3. Professional Instability

AVFs redefined the participants’ bodies as “bodies requiring management” within the professional world. The visible marks on the arm fostered the perception that exposure at work could lead to negative evaluations or job insecurity. Consequently, some participants continuously adjusted their clothing and behavior to conceal it while working under constant tension. However, in occupations that require physical activity, the conflict between protecting the AVF and maintaining a livelihood may ultimately lead to work cessation. In this process, the AVF functioned as a destabilizing factor in occupational identity, redefining the conditions of a “body capable of work” beyond its role as a therapeutic device.

“I hid it and worked, or else I’d get fired… If you wear gloves, they don’t notice, right… No one at the company knew for 10 years”.

(P6, 66 years)

“Look how bulgy it is. Even after all this time, mine hasn’t bulged out that much. It hasn’t bulged out this much. Even after 30 years, it hasn’t bulged out as much as other people’s”.

(P4, 70 years)

### 3.5. Acceptance Formed Within the Polarity of Life and Threat

For the participants, the AVF was not just a physical structure but also a conduit enabling survival and, simultaneously, a source of anxiety that it could be lost at any moment. Recognizing these conflicting meanings, they sought to accept the AVF in their own way.

#### 3.5.1. Regaining a Feeling of Control Through Comparison and Practice

Participants perceived the AVF as a part of their body that required constant awareness and management. Accordingly, they used various self-protective strategies at both psychological and physical levels. They sought relative reassurance by comparing their own AVFs with those of others while repeatedly applying daily management methods to reduce protrusion or prevent damage. This comparison and practice emerged as part of their efforts to understand and manage their altered physical states.

“Other people’s veins are really something… Some people have it really bad… I’m lucky it’s only this bad…”

(P4, 70 years)

“I never use a tourniquet… You have to know how to press with your hand and take care of your arm”.

(P6, 66 years)

#### 3.5.2. The Dual Meaning of an AVF

Participants perceived the AVF as an essential conduit for sustaining life. The visible blood vessels were both a mark of pain and illness and, at the same time, a symbol of reassurance that dialysis would always be available. However, the same marker also implied the possibility of losing function at any time, evoking the risks of further surgery and bodily harm. Thus, the AVF was experienced as both an enabler of survival and a source of anxiety, and participants lived with this simultaneous awareness of its contradictory meanings.

“Seeing the veins hurts my heart… But because of this, I’m keeping myself alive… I live comforting myself with that thought”.

(P4, 70 years)

“Thanks to this, I’ve lived this long. I don’t know how much longer it’ll last. If this fails, I’ll have to move it again. What if I can’t use either arm?”

(P8, 71 years)

## 4. Discussion

This study explored the experiences of women undergoing HD who underwent AVF formation, demonstrating that AVFs function not simply as vascular access points but as life-sustaining, visible markers that anchor the disease to the body’s surface. While previous studies focused on physiological outcomes, such as maintaining patency rates and averting complications, this study revealed how AVF visibility alters patients’ self-perception, relational positioning, and the structure of their daily lives. An AVF is both a means of treatment and a physical trace that visualizes illness, causing women to experience destabilization and repositioning. This perspective is consistent with prior research reporting that patient experiences with vascular access are deeply connected to quality of life and identity perception [20], while specifically stressing the “visible body.”

Participants experienced the AVF as an “alien body.” This replicates the situations described in Merleau-Ponty’s theory of embodiment [21], where the body, once naturally used in daily life, is no longer a transparent entity unconsciously perceived but rather an object that calls for attention and recognition. The protruding blood vessels transform the body from a transparent entity into a visible object, thus destabilizing self-perception. These results concur with those of previous qualitative research reporting an association between body image distortion and depression and anxiety in patients with CKD [22]. It has been reported that patients with CKD experience restrictions in daily activities and self-perception owing to changes in their physical condition and function [23]. These results demonstrate that bodily changes can affect an individual’s life structure and emotional adaptation beyond physiological issues. Furthermore, studies on HD patients have reported that anxiety, depression, and symptom burden are closely associated with reduced quality of life [24,25,26]. Notably, the higher sensitivity to changes in bodily appearance observed in women was consistent with the findings of this qualitative study and existing quantitative research. Furthermore, research shows that body image impairment is significantly associated with quality of life in patients with CKD [27]. This supports the notion that the visibility of an AVF is not simply a superficial change but a condition connected to the emotional and functional dimensions.

The sense of loss and recognition of a changed self, repeatedly expressed by the participants, corresponds to Bury’s concept of “biographical disruption” [28], which proposes that chronic illness disrupts the continuity of an individual’s life and forces a redefinition of existing identity. Biographical disruption signifies that illness is not simply a physical abnormality but an event that compels individuals to re-examine the flow of their lived experiences and their expectations for the future. In this study, the AVF served as a life-sustaining treatment device while simultaneously acting as a catalyst that continually prompted participants to contrast their prior selves with their present selves. This can be understood as the disease intervening not only in the body but also in the structure of self-perception.

An AVF is particularly distinctive as it involves visible changes on the skin’s surface, unlike internal functional decline. Such visibility compelled the participants to interpret their bodies through others’ gazes, leading to strategic behaviors such as concealment, avoidance of exposure, and restrictions on using public spaces. This is connected to Goffman’s concept of stigma [29], which argues that when physical differences acquire meaning in social interactions, individuals anticipate possible evaluations and adjust their appearance and behavior. In other words, the participants’ experiences can be understood not simply as an adaptation to physical functional changes but as a process of repositioning themselves by choosing how to reveal or control their altered bodies within social relationships. In addition to individual and relational experiences, the findings of this study should be understood within a broader sociocultural context. In many societies, including Korea, women’s bodies are subject to socially constructed expectations regarding appearance, attractiveness, and bodily integrity. These expectations are reinforced through media representations and social norms that emphasize physical appearance as an important component of feminine identity. Within this context, the visible bodily changes caused by the AVF may carry meanings beyond physical alteration, functioning as a socially interpreted marker that influences self-perception and social interactions. Participants’ experiences of concealment, avoidance, and sensitivity to others’ gazes can thus be understood not only as individual coping responses but also as reflections of internalized sociocultural expectations regarding the female body. These findings suggest that the experience of AVF in women should be interpreted within the intersection of illness, gender norms, and sociocultural context, highlighting the need for sex-sensitive and culturally informed approaches in clinical practice.

Recent qualitative studies exploring HD patients’ treatment experiences and disease perceptions have reported that the processes of interpreting and meaning-making around their condition are closely linked to psychological adaptation [30]. Furthermore, studies synthesizing the lived experiences of HD patients suggest that perceptions of illness and emotional effects are closely associated with quality of life and assimilation into the treatment process [30,31]. These prior studies support the possibility that the concealment strategies or relational avoidance observed in this research are not simply individual reactions but adjusted strategies formed within psychosocial contexts.

It also provides important insights into the repositioning of one’s place within the relational world. The transition to a protected role within the family, distancing oneself from peer groups, and the instability of one’s professional identity can be understood not as simple role changes but as processes of reconstructing relational identity. A recent extensive analysis of the lived experiences of HD patients also reported that they gradually experienced being redefined as protected or dependent individuals [31], and it was suggested that such changes could lead to undermined perceptions of autonomy and emotional withdrawal.

The ambivalent nature of AVFs is closely linked to the participants’ persistent uncertainty. While accepting the AVF as a key lifeline enabling survival, participants simultaneously acknowledged the potential threat of its failure at any time. This twofold perception resembles the uncertainty experienced by individuals when predictability is limited during the disease process. Indeed, recent studies have reported that disease-related uncertainty in HD patients significantly increases anxiety and depression levels and is associated with reduced quality of life [32].

This study illustrates that AVFs extend beyond the scope of mere therapeutic devices; they affect the overall lives of women undergoing HD. An AVF functions as both a bodily structure that enables survival and a visible marker of illness, consequently altering self-perception and relational positioning. This affects role performance within the family, peer, and occupational spheres, intersecting with appearance norms and caregiving expectations for women, and expressing itself as a rearrangement of life. Therefore, the AVF experience must be understood not simply as a dimension of treatment adaptation but as a reconfiguration of women’s lives through visible bodily changes.

These conclusions highlight the need to restructure AVF management strategies. While current vascular access management primarily focuses on physiological indicators, such as patency rate maintenance and complication prevention, this study found that AVFs also involve psychosocial factors that affect patients’ self-perception, relational positioning, and treatment compliance. Therefore, AVF management should not be limited to technical access maintenance but should be expanded into an integrated management model that includes early assessment of body image perception, disease-related uncertainty, and experiences of social stigma. Structured psychosocial screening and counseling interventions, particularly in the early stages of dialysis, can be considered preventive management strategies to improve treatment compliance and quality of life.

Furthermore, the findings of this study emphasize the need for a patient management approach that incorporates a sex-sensitive perspective within a multidisciplinary collaborative system (nephrology nurses, physicians, psychologists, and social workers). Women undergoing HD experience bodily changes within a cultural context in which these changes intersect with appearance norms and caregiving role expectations; thus, management strategies must also consider these sociocultural elements. Structured counseling on long-term vascular planning, education on coping with situations involving bodily exposure, and support for adjusting roles within family and workplace settings are vital aspects of safe and high-quality HD management. Ultimately, AVF management needs to be redefined not simply as procedural care but as an integrated system that supports patients throughout their lives. This was a qualitative investigation of women within a single cultural context, limiting its generalizability; furthermore, it also relied on self-reported data. Additional research should validate the effects of AVF visibility on life by incorporating sex comparisons and multiple data sources.

## 5. Limitations

This study has several limitations that should be considered when interpreting the findings.

First, this study was conducted with a relatively small sample of nine participants, which is consistent with the nature of phenomenological research but may limit the transferability of the findings. Although data saturation was achieved, the experiences captured may not fully represent all women undergoing HD with an AVF.

Second, participants were recruited from a specific region within South Korea, reflecting a single sociocultural context. Given that body image, gender roles, and social expectations are culturally constructed, the findings may not be directly applicable to different cultural or healthcare settings.

Third, the study relied on self-reported data obtained through in-depth interviews. While this approach is essential for capturing lived experiences, it may be subject to recall bias or social desirability bias.

Finally, this study employed a qualitative design without incorporating quantitative measures such as standardized assessments of quality of life, anxiety, or depression. Future studies using mixed-method approaches may provide a more comprehensive understanding of the psychosocial impact of AVF in women undergoing HD.

## 6. Conclusions

This study, through the experiences of women undergoing HD who underwent AVF formation, demonstrated that the AVF functioned not solely as a vascular access point but also as a life-sustaining, visible marker that simultaneously anchors the disease to the body’s surface. The visibility of the AVF affected patients’ body awareness and self-understanding, acting as a factor that readjusted their relational positions within their family, peer group, and professional world. That is, the AVF was experienced as both a therapeutic device and an entity that intervened in the structure of life. This study suggests that AVF management should not be limited to the preservation of physiological functions. Changes in body image, social gaze, and disease-related uncertainty may significantly affect treatment compliance and quality of life, highlighting the need to incorporate these factors into management strategies. Therefore, vascular access management systems should include initial psychosocial assessments, education on body image and role adjustment, and structured counseling support. In particular, a sex-sensitive approach that considers the sociocultural context of women can help build a more patient-centered, sustainable model of HD management.

## Figures and Tables

**Table 1:** Participant characteristics (N = 9).

Participant	Age (Years)	Marital Status	Education	Religion	Employment Status	Year of Hemodialysis (HD) Initiation
1	57	Widowed	High school	Christian	Unemployed	2008
2	79	Divorced	Middle school	Buddhist	Unemployed	2020
3	51	Single	High school	Buddhist	Unemployed	2022
4	70	Married	Middle school	Buddhist	Unemployed	2023
5	56	Divorced	High school	Buddhist	Unemployed	2020
6	66	Married	High school	Christian	Unemployed	2000
7	46	Married	High school	Buddhist	Self-employed	2023
8	71	Widowed	Middle school	None/Other	Unemployed	2021
9	64	Widowed	Middle school	Catholic	Unemployed	2004

**Table 2:** Essential structure of the lived experiences of women with arteriovenous fistula (AVF).

Core Theme	Theme Cluster	Theme	Subthemes
Women’s lives shaken and repositioned amid visible, life-sustaining bodily changes	Shaken self-confrontation of the visible body	Encountering the unfamiliar body	Anxiety due to physical changes after AVF formation Recurring feelings of loss Negative self-stigmatization
		The body experienced through others’ gazes	Concealment of the AVF Avoidance of exposure situations
	Being repositioned within the relational world	Readjustment of position within the family	Transition to a protected entity Renegotiation of roles with the patient’s spouse
		Distance in peer relationships	Changes in friendships Experiences of being marginalized
		Professional instability	The burden of maintaining employment Disruption of livelihood
	Acceptance formed within the polarity of life and threat	Regaining a feeling of control through comparison and practice	Psychological protective behaviors through AVF comparison Physical protective behaviors to prevent AVF changes
		The dual meaning of the AVF	Life-sustaining entity Threat-posing entity

## Data Availability

The data presented in this study are available on request from the corresponding author. The data are not publicly available due to ethical restrictions and the need to protect participant confidentiality.

## Supplementary Materials

The following supporting information can be downloaded at: https://www.mdpi.com/article/10.3390/healthcare14101296/s1, Table S1: Lived Experiences of Women with Arteriovenous Fistula Undergoing Hemodialysis: A Phenomenological Study.

## Abbreviations

The following abbreviations are used in this manuscript:

AVF	Arteriovenous fistula
HD	Hemodialysis
CKD	Chronic kidney disease
KRT	Kidney replacement therapy
